# Blood Biomarkers for Identifying Alzheimer's Disease Pathology in Dementia Patients

**DOI:** 10.1002/alz70856_102523

**Published:** 2025-12-25

**Authors:** Tianyi Wang, Yuyue Qiu, Li Shang, Jialu Bao, Yutong Zou, Yifei Wang, Yuhan Jiang, Bo Li, Yixuan Huang, Wenjun Wang, Longze Sha, Meiqi Wu, Shanshan Chu, Wei Jin, Dan Lei, Qi Xu, Li Huo, Liling Dong, Chenhui Mao, Jing Gao

**Affiliations:** ^1^ Peking Union Medical College Hospital, Beijing, Beijing, China; ^2^ Peking Union Medical College Hospital, Beijing, China; ^3^ Institute of Basic Medical Sciences & Neuroscience Center, Chinese Academy of Medical Sciences and Peking Union Medical College, Beijing, Beijing, China

## Abstract

**Background:**

To evaluate the diagnostic efficacy of plasma biomarkers in distinguishing Alzheimer's disease (AD) from a variety of other neurodegenerative dementias, using both SIMOA and Lumipulse as the detection methods for blood biomarker measurement.

**Method:**

A total of 292 patients were recruited from the Peking Union Medical College Hospital (PUMCH) dementia cohort. The clinical diagnosis of these patients includes Alzheimer's disease (AD), Cerebral amyloid angiopathy (CAA), Creutzfeldt‐Jakob disease (CJD), frontotemporal dementia (FTD), hereditary diffuse leukoencephalopathy with spheroids (HDLS), neuronal intranuclear inclusion disease (NIID), normal pressure hydrocephalus (NPH), vascular dementia (VAD) and dementia of undetermined etiology. Plasma biomarkers were measured using SIMOA and Lumipulse commercially available kits.

**Result:**

Plasma levels of Aβ42/40, pTau181, pTau217, GFAP, and NFL were compared across different clinical diagnostic groups of neurodegenerative diseases in dementia patients. Among these biomarkers, plasma pTau217 showed the most significant difference between AD and other neurodegenerative disease. However, in specific disease groups such as CAA and CJD, plasma pTau217 levels were elevated to levels comparable to those observed in AD. Results for all plasma markers correlated well between SIMOA and Lumipulse, with the strongest correlation observed for plasma pTau217 levels. Based on FDA‐approved CSF biomarkers, plasma pTau217 was able to predict CSF Aβ status with high accuracy over 0.9. We also compared plasma biomarker levels differences between CSF A‐T‐, A+T‐, A+T+ groups, no statistical significance was found in Aβ42/40, pTau181, pTau217, GFAP, and NfL biomarkers level between A+T‐ and A+T+ group, but a trend of plasma pTau217 elevation was shown. Finally, the plasma pTau217 cutoff value was applied to predict Aβ PET and tau PET classifications, in accuracy up to 0.977.

**Conclusion:**

Plasma pTau217 levels can effectively differentiate AD pathology in dementia patients, with accuracy comparable to that of CSF and PET imaging.

